# Immediate Versus Delayed Breast Reconstruction: Integrating Oncologic Safety, Adjuvant Therapy Considerations, and Aesthetic Outcomes in Multidisciplinary Breast Cancer Care

**DOI:** 10.7759/cureus.108405

**Published:** 2026-05-07

**Authors:** Sofia I Balamoti, Kyriaki Alevrogianni, Konstantinos Benetatos

**Affiliations:** 1 Breast Surgery, Biokliniki of Athens, Athens, GRC; 2 Surgery, 401 General Military Hospital of Athens, Athens, GRC; 3 Plastic and Reconstructive Surgery, 401 General Military Hospital of Athens, Athens, GRC

**Keywords:** aesthetic outcomes, breast cancer, breast reconstruction, delayed reconstruction, immediate reconstruction, mastectomy, multidisciplinary care, radiotherapy

## Abstract

Breast reconstruction has become an integral component of modern breast cancer management, aiming to restore body image and improve the quality of life following mastectomy. The optimal timing of reconstruction remains controversial, particularly in the context of adjuvant therapies such as radiotherapy. The aim of this review is to evaluate current evidence comparing immediate with delayed breast reconstruction, focusing on oncologic safety, interaction with adjuvant therapy, complication profiles, and aesthetic outcomes. A narrative review of the literature was performed, incorporating data from meta-analyses, prospective cohort studies, and multicenter trials assessing outcomes of immediate and delayed breast reconstruction. Immediate breast reconstruction is associated with improved aesthetic outcomes and reduced psychosocial distress while maintaining oncologic safety in appropriately selected patients. Evidence suggests that immediate reconstruction does not increase recurrence rates or negatively affect survival outcomes. However, higher complication rates may be observed, particularly in patients requiring postmastectomy radiotherapy. Delayed reconstruction provides greater flexibility in oncologic management and may reduce complication risk in selected patients, although it is often associated with less favorable aesthetic outcomes. Hybrid approaches such as delayed-immediate reconstruction have emerged to balance these competing considerations. The decision between immediate and delayed breast reconstruction should be individualized within a multidisciplinary framework. The integration of oncologic factors, anticipated adjuvant therapy, and patient preferences is essential to optimize both clinical and aesthetic outcomes.

## Introduction and background

Breast cancer remains the most frequently diagnosed malignancy among women worldwide, and advances in early detection and multimodal treatment (including surgery, systemic therapy such as chemotherapy or hormone therapy, and radiotherapy) have led to a steady improvement in survival over recent decades [1]. As survival continues to increase, the focus of care has progressively expanded beyond oncologic control to include the quality of life, body image, and long-term functional and aesthetic outcomes [2]. Within this evolving landscape, breast reconstruction has transitioned from a secondary consideration to an essential component of comprehensive breast cancer management [3].

Mastectomy, although oncologically effective, is associated with significant physical and psychological consequences, often affecting body image, femininity, and sexual well-being in a profound and lasting manner [2]. Reconstruction aims to mitigate these effects, and its beneficial impact on patient-reported outcomes has been consistently demonstrated, particularly in relation to psychosocial recovery and the restoration of self-perception [3]. As a result, both patient expectations and surgical practice have shifted toward a more integrated approach in which reconstructive planning is incorporated early in the treatment pathway rather than considered as a delayed option.

A critical component of this approach is the determination of the optimal timing of reconstruction, which remains one of the most complex and debated aspects of modern breast cancer care [4]. Immediate breast reconstruction, performed at the time of mastectomy, offers important advantages, including the preservation of the native skin envelope (the skin covering the breast preserved during mastectomy), the maintenance of breast contour, and the potential for superior aesthetic outcomes, while also reducing the psychological burden associated with the loss of the breast [5]. In contrast, delayed reconstruction, performed after the completion of oncologic treatment, provides greater flexibility in patients requiring adjuvant therapies (postoperative treatments such as chemotherapy, hormone therapy, or radiotherapy) and may reduce the risk of complications in selected clinical scenarios [6].

Despite the widespread adoption of both approaches, the choice between immediate and delayed reconstruction remains highly individualized and often controversial in clinical practice. Historically, concerns have been raised regarding the oncologic safety of immediate reconstruction, particularly in relation to the possibility of the delayed detection of local recurrence or interference with adjuvant treatment delivery. However, contemporary evidence suggests that, when appropriately selected, immediate reconstruction does not adversely affect recurrence rates or overall survival outcomes [7].

At the same time, the interaction between breast reconstruction and postmastectomy radiotherapy continues to represent a major point of tension in decision-making, as radiotherapy has been associated with increased complication rates and suboptimal aesthetic results, particularly in implant-based reconstruction [4]. These challenges are further amplified by the increasing complexity of breast cancer management, including the widespread use of neoadjuvant chemotherapy and the need for individualized multidisciplinary treatment planning. Recent large-scale studies have demonstrated that although immediate reconstruction may increase the interval between diagnosis and definitive surgery, it does not result in clinically meaningful delays in the initiation of adjuvant therapy, provided that postoperative complications are minimized [8].

Given these considerations, the decision regarding the timing of breast reconstruction cannot be reduced to a uniform algorithm but instead requires a tailored approach that balances oncologic factors, reconstructive goals, and patient expectations within a multidisciplinary framework. The development of hybrid strategies such as delayed-immediate reconstruction reflects the ongoing effort to reconcile these competing priorities and optimize both clinical and aesthetic outcomes [6]. The aim of the present review is to critically evaluate the current evidence comparing immediate with delayed breast reconstruction, with particular emphasis on oncologic safety, interaction with adjuvant therapies, complication profiles, and aesthetic outcomes, in order to provide a comprehensive framework for clinical decision-making.

## Review

Methods

This study was conducted as a narrative review of the literature examining the timing of breast reconstruction in patients undergoing mastectomy for breast cancer, with particular emphasis on the comparison between immediate and delayed reconstruction and their impact on oncologic and reconstructive outcomes [9]. The review was designed to provide a clinically oriented synthesis of current evidence, focusing on how reconstruction timing interacts with oncologic safety, adjuvant therapies, and patient-centered outcomes within the context of modern multidisciplinary breast cancer care. In contrast to strictly protocol-driven systematic reviews, a narrative approach was selected in order to allow the integration of heterogeneous data across different study designs and to reflect the complexity of real-world clinical decision-making.

Relevant studies were identified through a structured search of electronic databases, including PubMed and Medical Literature Analysis and Retrieval System Online (MEDLINE), which represent the primary repositories for peer-reviewed literature in reconstructive and oncologic surgery. The search strategy incorporated combinations of keywords, including breast reconstruction, immediate reconstruction, delayed reconstruction, mastectomy, radiotherapy, and oncologic outcomes [10]. The search was limited to English-language publications and was complemented by the manual review of reference lists from key articles in order to ensure the comprehensive coverage of the topic and the inclusion of influential studies that may not have been captured through database search alone.

Study selection was based on clinical relevance to the aims of the review rather than strict methodological inclusion and exclusion criteria, in keeping with the narrative design [11]. Priority was given to meta-analyses, systematic reviews, prospective cohort studies, and large retrospective series that provided clinically meaningful data on reconstruction timing, complication profiles, and oncologic outcomes [12]. Particular emphasis was placed on studies evaluating the impact of postmastectomy radiotherapy on reconstructive outcomes, as well as investigations examining patient-reported outcomes and the quality of life following immediate and delayed reconstruction [13]. Both implant-based and autologous reconstruction techniques were included in order to provide a comprehensive overview of current reconstructive strategies and their respective advantages and limitations across different clinical scenarios.

Data from the selected studies were synthesized qualitatively with the aim of identifying consistent patterns across the literature, as well as areas of controversy or ongoing debate [14]. In practice, emphasis was placed on how reconstruction timing influences surgical decision-making, oncologic management, and aesthetic outcomes while taking into account the heterogeneity of study designs, patient populations, and outcome measures. Given this variability, a formal quantitative meta-analysis was not performed, and a narrative synthesis approach was adopted to integrate findings in a clinically meaningful manner and to highlight key factors that inform decision-making in contemporary breast reconstruction.

Results

Immediate Breast Reconstruction

Immediate breast reconstruction, defined as reconstruction performed at the time of mastectomy, has become a central component of contemporary breast cancer care, reflecting a paradigm shift toward integrating oncologic resection and reconstructive restoration within a single operative setting [15]. This evolution is driven not only by advances in surgical techniques but also by a broader recognition of the importance of aesthetic and psychosocial outcomes in overall patient care [16]. The preservation of the native skin envelope and, in selected cases, the nipple-areolar complex allows for the more accurate restoration of breast contour, projection, and symmetry, which consistently translates into superior aesthetic outcomes when compared with delayed approaches [17]. However, these advantages must be interpreted within the context of patient selection, as the benefits of immediate reconstruction are not uniformly applicable across all clinical scenarios, particularly in patients with a high likelihood of requiring adjuvant radiotherapy.

Beyond aesthetic considerations, immediate reconstruction plays a significant role in psychosocial recovery. The avoidance of a prolonged period without a breast mound has been associated with improved body image and reduced psychological distress, particularly in the early postoperative period [18]. This early restoration of physical integrity appears to influence broader aspects of patient-reported outcomes, including emotional well-being and social functioning, as demonstrated in prospective studies using validated outcome measures [19]. Nevertheless, while these benefits support the increasing use of immediate reconstruction, they must be balanced against the potential risks associated with surgical complexity and postoperative complications.

From a technical standpoint, immediate reconstruction includes a spectrum of approaches ranging from implant-based reconstruction to autologous tissue transfer, most commonly using abdominal-based flaps such as the deep inferior epigastric perforator flap [20]. Implant-based reconstruction remains the most frequently performed technique due to shorter operative time and lower immediate surgical burden, whereas autologous reconstruction offers superior long-term durability and more natural aesthetic outcomes [21]. Advances in microsurgical techniques have significantly improved the reliability of autologous reconstruction, allowing its use in increasingly complex clinical settings and expanding its role within immediate reconstructive strategies [22].

Despite these advantages, immediate reconstruction is associated with specific limitations that must be carefully considered. Higher rates of early postoperative complications have been reported, particularly in implant-based reconstruction, including infection, seroma formation, and wound healing complications that may compromise reconstructive success [23]. Importantly, these complications represent the primary factor associated with delays in adjuvant therapy rather than the timing of reconstruction itself, suggesting that surgical outcomes rather than reconstructive timing drive oncologic timelines [24]. In parallel, autologous reconstruction is increasingly recognized as a reliable salvage option for managing complications following implant-based reconstruction, particularly in the context of radiotherapy-induced tissue damage [25].

Taken together, immediate breast reconstruction offers clear advantages in aesthetic and psychosocial outcomes while maintaining oncologic safety in appropriately selected patients. Its optimal application requires the careful integration of patient characteristics, tumor biology, and anticipated adjuvant therapies within a multidisciplinary framework, highlighting the importance of individualized decision-making rather than a uniform preference for immediate timing.

Delayed Breast Reconstruction

Delayed breast reconstruction remains an essential component of the reconstructive algorithm, particularly in patients with advanced disease, uncertain oncologic margins, or a high likelihood of requiring postmastectomy radiotherapy [26]. By deferring reconstruction until the completion of oncologic treatment, this approach prioritizes cancer management and allows reconstructive planning to occur in a more stable clinical environment. This temporal separation between oncologic and reconstructive procedures provides flexibility in treatment sequencing and may reduce the risk of complications associated with operating in the setting of active adjuvant therapy.

One of the principal advantages of delayed reconstruction is its association with lower complication rates in selected patient populations, particularly in implant-based reconstruction [27]. Large prospective and multicenter studies have demonstrated reduced rates of overall and major complications when reconstruction is performed after the completion of initial oncologic treatment, an effect that is especially relevant in patients with significant comorbidities or compromised tissue quality [28]. Furthermore, delayed reconstruction allows for a more accurate assessment of the long-term effects of radiotherapy, facilitating the selection of the most appropriate reconstructive technique based on the final tissue environment.

However, these advantages must be weighed against the inherent limitations of delayed reconstruction. The absence of a preserved skin envelope following mastectomy often necessitates more complex reconstructive strategies, including staged procedures and extensive tissue manipulation, which may compromise aesthetic outcomes when compared with immediate reconstruction [29]. In addition, the reconstruction of previously irradiated tissues is associated with increased technical difficulty due to fibrosis, reduced vascularity, and impaired wound healing, all of which may negatively impact both complication rates and long-term results [30].

From a patient-centered perspective, delayed reconstruction is associated with a prolonged period without a breast mound, which has been shown to adversely affect body image and psychological well-being [31]. This interval may represent a significant source of distress and has been identified as a key factor influencing patient preference toward immediate reconstruction when oncologically feasible [32]. Moreover, delayed reconstruction often involves extended treatment timelines and multiple procedures, which may further influence patient experience and overall satisfaction.

Despite these limitations, delayed reconstruction remains a valuable and often necessary option in complex oncologic scenarios. Its role continues to evolve as advances in reconstructive techniques and multidisciplinary care improve the ability to tailor treatment strategies to individual patient needs, reinforcing its importance as part of a comprehensive reconstructive approach.

Impact of Radiotherapy on Reconstruction Timing

The interaction between breast reconstruction and postmastectomy radiotherapy represents one of the most critical determinants of reconstructive timing and remains a central challenge in clinical decision-making [33]. Radiotherapy has a well-documented impact on both complication rates and aesthetic outcomes, particularly in implant-based reconstruction, where radiation-induced fibrosis, vascular damage, and impaired wound healing contribute to higher rates of capsular contracture, implant loss, and suboptimal cosmetic results [34].

These effects have historically led to caution in performing immediate reconstruction in patients likely to require radiotherapy, particularly when implant-based techniques are considered. However, contemporary evidence suggests that this relationship is more nuanced, particularly in the context of autologous reconstruction [35]. Although radiotherapy may still result in fat necrosis, volume loss, and changes in tissue quality, autologous flaps appear to be more resilient to its effects, maintaining acceptable complication rates and long-term outcomes in many cases [36].

This evolving understanding has led to the development of hybrid strategies such as delayed-immediate reconstruction, which aim to combine the advantages of both immediate and delayed approaches [37]. By preserving the skin envelope at the time of mastectomy and delaying definitive reconstruction until after the completion of radiotherapy, this strategy provides flexibility in oncologic management while maintaining the potential for improved aesthetic outcomes. Decision-making in this setting requires the careful assessment of tumor characteristics, the likelihood of radiotherapy, and patient-specific factors, emphasizing the importance of multidisciplinary collaboration in optimizing outcomes.

Clinical Outcomes and Complications

Clinical outcomes following breast reconstruction are influenced by a complex interplay of factors, including reconstruction timing, surgical technique, patient characteristics, and adjuvant therapies [38]. Both immediate and delayed reconstruction can achieve satisfactory outcomes when appropriately selected, although important differences exist in complication profiles, aesthetic results, and patient-reported satisfaction.

Aesthetic outcomes are generally more favorable in immediate reconstruction, largely due to the preservation of the native skin envelope and the more accurate restoration of breast contour, projection, and symmetry. In contrast, delayed reconstruction is often performed in a setting of tissue deficiency or post-radiotherapy changes, which may necessitate more complex reconstructive strategies and may limit the predictability of cosmetic results. Despite these differences, long-term aesthetic refinement is frequently achievable in both approaches through staged procedures such as fat grafting and contralateral symmetrization, highlighting the dynamic nature of reconstructive outcomes over time [29].

Complication rates vary significantly depending on both reconstructive modality and clinical context. Implant-based reconstruction is associated with higher rates of complications in the setting of radiotherapy, including capsular contracture, infection, and implant loss, whereas autologous reconstruction demonstrates greater resilience to radiation-related changes but carries its own risks related to donor-site morbidity and longer operative times [30]. Early postoperative complications, particularly wound healing issues and infection, remain a critical determinant of overall outcomes, as they may compromise reconstructive success and potentially delay the initiation of adjuvant therapy [31].

From an oncologic perspective, contemporary evidence supports the safety of both immediate and delayed reconstruction, with no consistent differences observed in recurrence rates or overall survival when appropriate patient selection criteria are applied [36]. However, variability across studies, differences in patient populations, and heterogeneity in outcome reporting continue to limit direct comparisons, underscoring the need for individualized decision-making rather than a uniform reconstructive strategy.

Patient-reported outcomes represent a central component of overall success and have been shown to correlate strongly with both aesthetic results and psychosocial recovery. Immediate reconstruction is often associated with improved early postoperative satisfaction and body image, whereas delayed reconstruction may be associated with a longer psychological adjustment period due to the interval without a breast mound [32]. Nevertheless, long-term satisfaction can be high in both groups, particularly when expectations are appropriately managed and reconstructive goals are aligned with patient preferences.

Overall, successful breast reconstruction depends on a tailored approach that integrates oncologic considerations, reconstructive technique, and patient-specific factors within a multidisciplinary framework. Rather than favoring a single timing strategy, current evidence supports a patient-centered model in which reconstruction is individualized to optimize both clinical and aesthetic outcomes across the continuum of breast cancer care.

Discussion

The findings of the present review suggest that the timing of breast reconstruction should not be approached as a simple dichotomous choice between immediate and delayed strategies but rather as a dynamic clinical decision that is inherently shaped by oncologic priorities, reconstructive considerations, and patient expectations within the broader context of modern breast cancer care [11]. While both approaches have consistently demonstrated oncologic safety when applied in appropriately selected patients, their relative advantages are best understood in terms of how effectively they integrate into the overall treatment pathway rather than as competing techniques in isolation [14]. This perspective reflects a broader evolution in breast surgery, where reconstruction is increasingly incorporated into primary treatment planning and coordinated across surgical and oncologic disciplines in order to optimize both survival and quality of life outcomes [3].

A further dimension that has gained increasing relevance is the role of nipple-sparing mastectomy within the setting of immediate reconstruction, as this technique has significantly expanded the potential for aesthetic preservation without compromising oncologic safety [5]. Large contemporary series have demonstrated low rates of local recurrence and acceptable complication profiles, including nipple necrosis rates as low as approximately 1.2% in cohorts exceeding 3,000 reconstructions, alongside consistently low recurrence rates across analyses involving more than 7,000 patients [6]. In addition, long-term data from high-volume centers suggest that nipple-sparing approaches, when combined with immediate reconstruction, can achieve superior aesthetic outcomes and high levels of patient satisfaction, reinforcing their role within modern reconstructive algorithms [7].

A key observation across contemporary studies is the consistent association of immediate reconstruction with superior early aesthetic and psychosocial outcomes, largely attributable to the preservation of the native skin envelope and the avoidance of a prolonged period without a breast mound [26]. This early restoration of body image appears to exert a meaningful influence on patient-reported outcomes, particularly in domains related to self-perception, emotional well-being, and social functioning, which are increasingly recognized as central endpoints in breast cancer care [27]. However, these advantages must be interpreted alongside the increased technical complexity of immediate reconstruction and the higher susceptibility to early postoperative complications, particularly in implant-based approaches where wound healing and infection remain relevant concerns [31]. Importantly, these complications represent the primary driver of delays in adjuvant therapy rather than reconstruction timing itself, emphasizing that surgical quality remains the key determinant in preserving oncologic treatment timelines [24].

The distinction between implant-based and autologous reconstruction represents a critical factor that intersects with timing decisions and has important implications for both short-term and long-term outcomes [10]. Large population-based analyses, including the Swedish Breast Reconstruction Outcome Study (SweBRO) 3 study, have demonstrated that autologous reconstruction is associated with higher long-term satisfaction and more stable aesthetic outcomes compared with implant-based techniques, particularly in patients exposed to radiotherapy [10]. Procedure-specific data, including deep inferior epigastric artery perforator (DIEP) flap analyses, further support that while autologous reconstruction involves increased operative complexity, it maintains acceptable complication rates and improved long-term durability, supporting its role in selected patient populations [11].

The interplay between reconstruction and radiotherapy continues to represent one of the most challenging aspects of decision-making, given the profound and often unpredictable effects of radiation on tissue quality and long-term reconstructive outcomes [2]. Implant-based reconstruction is particularly vulnerable to radiation-induced complications, including capsular contracture, implant exposure, and reconstructive failure, which may ultimately require revision or conversion to autologous techniques [34]. In contrast, autologous reconstruction appears to better tolerate radiation-related tissue changes, maintaining more stable long-term outcomes despite potential issues such as fat necrosis or volume loss [30]. These differences highlight the importance of anticipating radiotherapy at the time of initial surgical planning, as the inadequate integration of adjuvant treatment considerations remains a key contributor to suboptimal reconstructive outcomes [35].

Within this context, the emergence of hybrid approaches such as delayed-immediate reconstruction reflects an effort to reconcile the competing priorities of oncologic safety and aesthetic preservation [6]. By preserving the skin envelope at the time of mastectomy and deferring definitive reconstruction until after the completion of adjuvant therapy, this strategy allows for flexibility in treatment sequencing while maintaining structural advantages associated with immediate reconstruction [33]. Although not universally applicable, such approaches illustrate the shift toward adaptive reconstructive pathways that respond to the clinical course of each patient rather than relying on rigid timing algorithms [22].

An additional layer of complexity arises from the increasing use of neoadjuvant chemotherapy, which alters both tumor biology and treatment sequencing in a way that directly impacts reconstructive planning [2]. Emerging evidence indicates that reconstruction following neoadjuvant therapy can be performed safely without compromising oncologic outcomes, with reported five-year local recurrence-free survival rates approaching 96%-97% and no significant differences in distant metastasis-free survival even in patients with locally advanced disease [12]. These findings reinforce the need to incorporate systemic therapy considerations early in the decision-making process, as they influence not only feasibility but also the optimal timing and type of reconstruction.

Ultimately, the comparison between immediate and delayed reconstruction is best understood as a context-dependent clinical decision shaped by oncologic factors, reconstructive strategy, and patient priorities, recognizing that both approaches can achieve excellent outcomes when applied judiciously [38]. The variability observed across studies reflects the heterogeneity of patient populations and treatment pathways, underscoring the importance of individualized planning supported by multidisciplinary expertise and informed patient involvement [21]. As reconstructive techniques continue to advance and patient-reported outcomes gain increasing importance, future research should focus on refining selection criteria and improving strategies that minimize complications while optimizing both aesthetic and functional results [28].

Emerging advances, including prepectoral implant placement, acellular dermal matrices, robotic nipple-sparing mastectomy, and the expanding role of fat grafting, further illustrate the rapidly evolving reconstructive landscape and are likely to influence future strategies in reconstruction timing and technique [35]. Prepectoral implant placement, for example, has been associated with reduced capsular contracture rates and improved soft-tissue dynamics, which may be particularly relevant in patients requiring radiotherapy and could shift decision-making toward immediate reconstruction in selected cases [35]. Similarly, robotic and minimally invasive approaches to nipple-sparing mastectomy may expand eligibility to patients previously considered suboptimal candidates, thereby increasing the feasibility of combining oncologic safety with enhanced aesthetic preservation [35].

Beyond clinical outcomes, cost-effectiveness and healthcare resource utilization represent increasingly important considerations in reconstructive decision-making [8]. Comparative analyses have demonstrated that staged reconstruction may incur substantially higher total costs compared with immediate approaches, with reported figures of approximately $106,000 versus $76,000, respectively, highlighting the potential long-term economic advantage of immediate reconstruction in selected settings [9]. It should be noted, however, that most available cost data are derived from the US healthcare system and may not be directly generalizable to other healthcare settings with different reimbursement structures and resource allocation models [8].

Despite the growing body of literature supporting both immediate and delayed breast reconstruction, several important limitations must be acknowledged when interpreting the available evidence. A substantial proportion of studies remain retrospective and therefore subject to selection bias, as patients undergoing immediate reconstruction are often younger, healthier, and treated in specialized centers, factors that may independently influence outcomes [21]. Considerable heterogeneity also exists across studies in terms of patient populations, reconstructive techniques, and outcome measures, limiting the ability to perform direct comparisons and draw definitive conclusions [28]. The relative scarcity of randomized controlled trials further constrains the strength of available evidence, particularly in relation to long-term oncologic outcomes and patient-reported measures of satisfaction and quality of life [11]. Variability in complication reporting and aesthetic assessment introduces additional challenges in synthesizing results across the literature [16]. In addition, important gaps remain in areas such as nipple-sparing mastectomy, cost-effectiveness analyses, and reconstruction in the setting of neoadjuvant chemotherapy, where high-quality prospective data are still limited, particularly outside of high-volume centers and specific healthcare systems [22].

## Conclusions

In conclusion, the timing of breast reconstruction after mastectomy should be viewed as a nuanced clinical decision rather than a binary choice between immediate and delayed approaches. Current evidence supports the oncologic safety of both strategies when appropriate patient selection is applied. Reconstruction is now considered an integral component of breast cancer care, requiring early multidisciplinary coordination to optimize outcomes. Immediate reconstruction offers advantages in preserving native anatomy and supporting early psychosocial recovery, though it carries a risk of postoperative complications that may impact adjuvant therapy. Delayed reconstruction remains essential in complex cases, particularly when postmastectomy radiotherapy is anticipated, providing flexibility in oncologic management. However, it may involve greater technical challenges and less predictable aesthetic outcomes. Radiotherapy continues to play a critical role in shaping both timing and technique due to its impact on complications and long-term results. This has driven a shift toward individualized, patient-specific surgical planning. Hybrid approaches such as delayed-immediate reconstruction further highlight the importance of adaptability in treatment strategies. Ultimately, optimal outcomes depend on personalized decision-making, multidisciplinary collaboration, and the meaningful incorporation of patient preferences.
